# Studyholism as a New Potential OCD-Related Disorder: What Evidence Have We Gathered until Now? A Narrative Review

**DOI:** 10.3390/bs14080684

**Published:** 2024-08-06

**Authors:** Yura Loscalzo

**Affiliations:** Department of Health Sciences, School of Psychology, University of Florence, 50135 Florence, Italy; yura.loscalzo@gmail.com

**Keywords:** studyholism, study addiction, workaholism, work addiction, study, study engagement

## Abstract

In 2017, Loscalzo and Giannini introduced the new potential clinical condition of studyholism (or obsession toward study) and a comprehensive model including its possible antecedents and outcomes. Then, emphasizing the value of avoiding an aprioristic (addiction) framework in analyzing problematic overstudying, they suggested conducting research on this new construct to unveil its internalizing and/or externalizing nature while also avoiding the over-pathologizing of a common behavior such as studying. Seven years after the first publication about studyholism, growing evidence concerning its antecedents suggested that studyholism might be defined as an OCD-related disorder (or, more generally, as an internalizing disorder). Moreover, the research about its outcomes highlighted that it is a problem behavior deserving attention as it is associated with academic, psychological, physical, and social downsides. Therefore, this paper aims to review the scientific literature published concerning studyholism to illuminate if it might be conceptualized as an OCD-related disorder based on its symptomatology, antecedents, and impact on individuals’ academic, physical, and psychological functioning. Given that it is a new construct, it is of critical value to systematize the findings gathered until now as it can help scholars interested in students’ well-being to have a clear understanding concerning the importance of screening studyholism since childhood, as this will help favor academic success and well-being and reduce the risk for school dropout. Finally, this paper presents an agenda for future research on studyholism, and it highlights the importance of further analyzing problematic overstudying using different theoretical perspectives (such as the behavioral addiction conceptualization) to unveil its real nature.

## 1. Introduction

Loscalzo and Giannini [1] coined and introduced the term “studyholism” in 2017 through a chapter that has been indexed on Scopus—following its quality assessment—in February 2019 (differently from what Loscalzo and Giannini [2] wrote before). More specifically, reviewing the wide literature about workaholism [3]—which is a condition characterized by problematic overworking and was first introduced in the 1970s by Oates [4]—Loscalzo and Giannini [3] noticed many different instruments for its assessment, which hindered the possibility of cumulative knowledge, as there was evidence that these instruments refer to different constructs [5,6]. Also, Loscalzo and Giannini [1] observed that only a few scholars analyzed workaholism in the school context, using a workaholism measure in student samples [7] or changing the term “work” with “study” into one of the instruments widely used for assessing workaholism within the behavioral addiction framework [8,9]. Loscalzo and Giannini [1] assumed that problematic overworking might take the form of problematic overstudying in students, considering that study is the primary work activity for students, as work is for workers. Hence, they proposed a comprehensive conceptualization of the new construct of studyholism, with the proclaimed aim to prevent studyholism from becoming (like workaholism) a confusing construct with various definitions and instruments that refer to different constructs because this would impair a coherent and unified understanding.

Seven years after the first publication concerning studyholism [1], there has been a growing number of studies supporting Loscalzo and Giannini’s conceptualization of studyholism as an OCD-related disorder. At the same time, there have also been publications using the term “studyholism” but referring to different measures and/or theoretical definitions [10,11,12]. More specifically, Salmela-Aro and Upadyaya [10]—next referenced by Yang et al. [11]—described studyholism as “the negative aspect of school engagement” (p. 950) and evaluated it with three items (instead of a validated instrument) whose Cronbach’s α sum score was 0.66. Alshammari et al. [12], instead, used the Studyholism Inventory (SI-10) by Loscalzo and Giannini [13] and also reported good alpha values for their sample [12]; however, the authors repeatedly define studyholism as a behavioral addiction (against Loscalzo and Giannini’s theorization [1]) throughout the paper. Moreover, the literature about problematic overstudying includes research that adopts the behavioral addiction framework. Like for studyholism, study addiction research is also developing [14,15,16,17,18,19]. However, study addiction papers do not always cite studyholism theorization [20,21,22] and this might increase confusion around problematic overstudying. 

Therefore, in order to prevent studyholism from facing the same fate as workaholism, systematizing the studyholism literature published so far is critical. Hence, this paper, after presenting Loscalzo and Giannini’s [1] conceptualization—by also highlighting the difference with study addiction [9]—will review the findings provided by the studyholism studies published so far, including the publications concerning its scales of measurement [13,23,24]. More specifically, through this comprehensive review, this paper aims to answer the following question: might studyholism be conceptualized as an OCD-related disorder based on its symptomatology, antecedents, and impact on individuals’ academic, physical, and psychological functioning? As explained in detail in the next paragraph, Loscalzo and Giannini [1], to avoid a priori assumptions, first theorized studyholism as potentially being characterized by both obsessive and addictive features. Then, based on their preliminary results [23], they suggested studyholism to be more like a study-related obsession rather than a behavioral addiction. Therefore, as later pointed out in their research papers, it is vital to further analyze this new potential clinical condition to unveil its obsessive and/or addictive nature through more research data. Thus, by systematizing the research about studyholism, this paper will shed light on the appropriateness of the OCD-related conceptualization for defying problematic overstudying, given that currently, in the scientific literature, there are two divergent operationalizations of this study-related condition: studyholism [1] and study addiction [9]. This paper does not aim to prove that the OCD-related conceptualization is better than the behavioral addiction one. Instead, it aims to provide thorough and systematized research-based evidence for the appropriateness of OCD-related theorization. In fact, to reach a firm conclusion about its obsessive and/or addictive nature—or, more generally, about its internalizing and/or externalizing nature—more research is still needed from both theoretical perspectives.

## 2. Studyholism: The Comprehensive Model by Loscalzo and Giannini

Concerning the term for defining problematic overstudying according to their conceptualization, Loscalzo and Giannini [1] chose “studyholism” since—not including the word “addiction”—it helps avoid reducing problematic overstudying to only externalizing/addiction features and negative/clinical aspects. In fact, Loscalzo and Giannini [1] stressed the importance of considering both externalizing/addiction and internalizing/obsessive features, as well as the possible co-presence of study engagement in students with problematic overstudying. Finally, given the aim of preventing studyholism from becoming a confusing construct with various definitions used interchangeably—while actually referring to different constructs—Loscalzo and Giannini [1] also felt it necessary to have a different name than the one used by Atroszko et al. [9]. Atroszko et al. [9], in fact, previously introduced the construct of “study addiction”, conceptualizing it as a behavioral addiction characterized by the seven core components of substance addictions [25]: salience, tolerance, mood modification, relapse, withdrawal, conflict, and problems. Moreover, Atroszko et al. [9] adapted the Bergen Work Addiction Scale (BWAS) [26] for its assessment through the change in the items of the words related to work with terms related to the study. They thus introduced the Bergen Study Addiction Scale (BStAS) [9].

As later clarified by Loscalzo and Giannini [2] in response to Atroszko’s commentary [27]—besides the fact that they coined the term “studyholism” [1] while working on its conceptualization before Atroszko et al.’s [9] publication —it is imperative to use different names for studyholism and study addiction as “doing so allows some clarity in the problematic overstudying literature and helps avoid the confusion surrounding the workaholism literature, which has been characterized by many different definitions under the umbrella terms workaholism and work addiction (usually used inter-changeably)” (pp. 428–429). More specifically, with regard to the raised criticism concerning the use of a term recalling alcoholism [27], they further explained—in line with what they previously stated [1]—that the term “studyholism” better represents their theory as it is a more generic term. It allows for the maintenance of a continuity with the workaholism literature—from which the construct originates—while also avoiding a reduction to only negative and addiction symptoms [2]. Moreover, Loscalzo and Giannini [2] highlighted that their approach is in line with Griffiths et al. [28]’s preference for the term “work addiction” to “workaholism.” Workaholism is a more generic term compared to work addiction, which emphasizes the addiction framework. 

Regarding the definition and operationalization of studyholism, Loscalzo and Giannini [1] followed Kardefelt-Winther’s [29] suggestions for the proposal of new potential behavioral addictions. He suggests [29] to go beyond a priori assumptions about addiction to identify the actual manifestation of problematic behaviors. Therefore, by also referring to their previous review of the workaholism literature [3], Loscalzo and Giannini [1] suggested an early definition of studyholism as a new potential clinical condition that includes both externalizing (i.e., addiction) and internalizing (i.e., obsessive-compulsive) symptoms, and either a low or high level of study engagement. They specified that study engagement is a positive attitude toward studying, including vigor, dedication, absorption [30], and intrinsic motivation for studying [1]. Then, to test this preliminary theorization, Loscalzo, Giannini, and Golonka [23] developed a pool of 68 items covering the three hypothesized facets of studyholism: obsessive-compulsive symptoms, addiction symptoms, and (high or low) study engagement. Their analyses on 340 Italian students suggested that studyholism does not include addiction features [23]. Therefore, Loscalzo and Giannini [1] polished their theorization and concluded that studyholism is neither a behavioral addiction nor a condition including both externalizing and internalizing symptoms (as they initially hypothesized using the “open approach” suggested by Kardefelt-Winther [29]). Hence, they speculated that studyholism is *more similar* to a study-related obsession than a study-related addiction. However, they also pointed out—as clearly stated in their theoretical paper concerning the comparison between the DSM-5 diagnostic criteria for OCD and Substance Use Disorder [31]—that no firm “conclusion can be made yet because of the lack of research on the specific features of problematic overstudying” (p. 869). 

From the inclusion of study engagement in the definition of studyholism, a distinction between engaged and disengaged studyholics arises. They both have high levels of studyholism; however, engaged studyholics also have high study engagement, while disengaged studyholics have low study engagement levels [1]. Regarding this distinction, Loscalzo and Giannini [1] initially suggested that the clinical form of studyholism is the disengaged type. However, they specified it is essential to address engaged studyholics to avoid their development into the disengaged type [1]. Based on the workaholism literature [32], in fact, they speculated that engaged studyholics are less impaired than disengaged studyholics. Moreover, they stated that it is crucial to consider overstudying as a pathological behavior only if it is associated with high functional impairment and low study engagement to avoid over-pathologizing studying, which is a common and usually positive behavior [33]. However, their subsequent research data highlighted that engaged studyholics should also be regarded as a clinical form. Disengaged studyholics are more impaired in the academic and affective areas but less in the social areas than engaged studyholics. Moreover, there is no difference between the two types of studyholism in terms of sleep quality, daytime sleepiness, stress, and aggressive behaviors in the university context [34]. Hence, according to their data, Loscalzo and Giannini [34]—as thoroughly presented in the next paragraph—suggested modifying their preliminary conceptualization by removing the reference to addiction symptoms and establishing two specifiers for studyholism: (i) study engagement level (high, average, low) and (ii) area of functional impairment (academic, social, both).

### 2.1. Studyholism: Its Current Operationalization

As demonstrated in the preceding paragraph, in their conceptualization of studyholism, Loscalzo and Giannini used an *open approach to research data*. Based on a thorough review of the workaholism literature [3], they suggested a preliminary theoretical model of studyholism. This model was not set in stone but instead designed to be refined based on data collected through rigorous scientific standards, devoid of confirmatory bias [1,24]. 

Hence, after conducting a study to test their central theoretical points [34], they defined studyholism as a new potential clinical condition characterized by high levels of obsessive-compulsive symptoms or an OCD-related disorder, which might also be associated with high study engagement levels [24]. Moreover, they also proposed the studyholism DSM-like criteria, specifying that they are tentative. These criteria should be tested using quantitative and qualitative studies; hence, criteria should be added or deleted based on the results gathered. Loscalzo and Giannini [24] designed the DSM-like criteria by referring to three sources: (i) their workaholism DSM-like criteria [3]; (ii) the DSM-5 OCD criteria [35]; and (iii) Loscalzo and Giannini’s [34] studyholism results, which were obtained from the unique research conducted at the time of Loscalzo and Giannini’s [24] paper. In summary, criterion A states that studyholism is characterized by persistent and recurrent problematic studying behaviors leading to clinically significant impairment or distress (including health-related problems) as revealed by the student displaying, for a minimum of 6 months, study-related obsessions and/or study-related compulsions. Moreover, as previously suggested by Loscalzo and Giannini [1], criteria B and C specify that studyholism symptoms should not be attributable to the physiological effects of a substance or another medical condition (criterion B) or be better explained by the symptoms of another mental disorder—such as the Obsessive-Compulsive Disorder, the Obsessive-Compulsive Personality Disorder, or Social Anxiety Disorder (criterion C). Finally, the diagnosis should be supplemented by two specifiers: (i) levels of study engagement (allowing for the distinction between engaged and disengaged studyholism) and (ii) area of impairment (academic, social, both). 

While this is the current definition and operationalization of studyholism, it is crucial to underscore that the literature on problematic overstudying (a broad term Loscalzo and Giannini used to encompass both studyholism and study addiction, without specific conceptualization [31]) is still in its infancy. More research is needed to arrive at a definitive definition of this potential clinical condition as an OCD-related disorder or a behavioral addiction. However, for those interested in studying problematic overstudying, it is imperative to consider the significant differences—as detailed in the next section—between studyholism and study addiction to avoid using the two constructs interchangeably.

### 2.2. The Differences between Studyholism and Study Addiction

Within the literature, two distinct conceptualizations of problematic overstudying have emerged: studyholism [1] and study addiction [9]. Loscalzo and Giannini [1] have undertaken the task of elucidating the primary disparities between these two constructs. While both share a common focus on the behavior of overstudying, they diverge in several significant ways.

First, while Loscalzo and Giannini [1] explicitly considered externalizing and internalizing features in their preliminary definition of studyholism, Atroszko et al. [9] only included externalizing features. More specifically, concerning internalizing symptoms, cognitive salience (introduced as an addiction component in the BStAS [9]) somehow encompasses obsessions [1]. However, as later better clarified by Loscalzo and Giannini [2], this internalizing feature has been introduced by Atroszko et al. [9] as an addiction/externalizing component (not as an internalizing feature); moreover, while study addiction has been suggested to be linked with internalizing features (such as perfectionism), the conceptualization of studyholism suggests instead that obsessive/internalizing symptoms are an intrinsic component of the construct. 

Another critical difference concerns the theoretical framework: study addiction used the behavioral addiction one, while Loscalzo and Giannini adopted the Heavy Study Investment (HSI) framework. In particular, Loscalzo and Giannini [1] adapted the Heavy Work Investment definition [36] to the study field and defined HSI as a heavy investment of time and effort in studying. This is a critical point of their theorization as it allows them to specify that while all studyholics are Heavy Study Investors (HSIs), not all HSIs are studyholics. Hence, by crossing studyholism and study engagement levels, Loscalzo and Giannini [1] suggested three types of HSIs: (i) engaged students (high levels of study engagement and low levels of studyholism); (ii) engaged studyholics (high levels of both studyholism and study engagement); and (iii) disengaged studyholics (high levels of studyholism and low levels of study engagement). Finally, by crossing the two facets, Loscalzo and Giannini [1] also introduced the “detached student”, who is not an HSI, as he/she has low studyholism and study engagement levels.

Finally, as apparent from the distinction between engaged and disengaged studyholics, Loscalzo and Giannini suggest that some students also have a positive study attitude jointly with studyholism. In contrast, the conceptualization of study addiction does not consider study engagement (or other positive aspects) in its definition [1]. Also, while Loscalzo and Giannini [1] suggest two subtypes of studyholism, Atroszko et al. [9] do not foresee subtypes. 

### 2.3. Studyholism: Antecedents and Outcomes 

**Figure 1:**
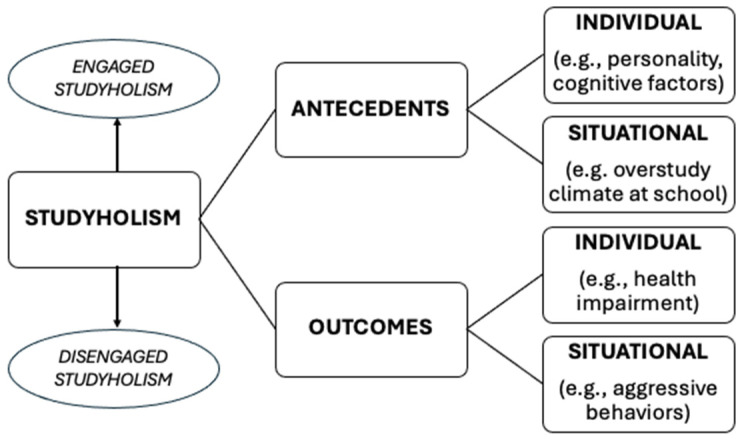
The studyholism comprehensive model by Loscalzo and Giannini [1].

Loscalzo and Giannini [1], in their seminal work, not only defined studyholism but also proposed a comprehensive model that includes potential antecedents and outcomes. Given the scarcity of research on problematic overstudying, they drew from their comprehensive model of workaholism [3] and suggested a classification for studyholism antecedents and outcomes into individual and situational [1]. They underscored the crucial need to differentiate between engaged and disengaged studyholics in studying studyholism antecedents and outcomes, and in analyzing the interaction between individual and situational factors regarding antecedents [1]. Figure 1 graphically summarizes the model.

About individual antecedents, Loscalzo and Giannini [1] listed personality traits (i.e., conscientiousness, neuroticism, openness to experience), perfectionism, motivation, cognitive factors (e.g., performance-based self-esteem or the tendency to continue working until having reached the feeling to have performed enough [37,38,39]), inability to down-regulate negative mood, and psychiatric disorders. They included the overstudying climate in the family and school settings for situational antecedents. In line with Mazzetti et al.’s [40] definition of overworking climate, Loscalzo and Giannini [1] introduced the overstudy climate to refer to students’ perception that their school and/or family expect an overstudy behavior from them. Hence, they speculated that high schools might have a higher overstudy school climate compared to technical and professional schools and that, at university, some majors might foster a higher overstudy climate (e.g., medical studies). Also, they suggested that students in their last year of study might experience a higher overstudy climate than in their first years, as they are closer to their final exams. Regarding family—in line with Kravina et al.’s [41] study, which showed that workaholic fathers foster workaholism in their sons—Loscalzo and Giannini [1] posited that an overstudying climate might be spread in the family as well.

Concerning individual outcomes, they listed low well-being at school, low academic performance, physical and psychological health impairment, and family functioning problems. For situational outcomes, they enumerated aggressive behaviors and low positive relationships in class [1]. Moreover, Loscalzo and Giannini [1] pointed out that school-related outcomes (i.e., low well-being at school and low positive relationships in class) might be especially evident in non-university contexts. There are fewer students at lower school levels than in university classes; therefore, there are usually closer relationships with peers and teachers than in universities. Hence, group dynamics could be more evident, and the impact of studyholics on the class might be more elevated. Finally, non-university students are obligated to attend school (in contrast with the university, where attendance is generally voluntary), with a potential higher impairment for studyholics who do not feel well in the school context [1].

## 3. A Review of the Studyholism Literature

In this section, the studyholism literature is reviewed with regard to the following topics: (i) the available scales to measure studyholism; (ii) the prevalence of studyholism; (iii) demographic and study-related differences in studyholism; (iv) studyholism outcomes, with particular reference to the impairment in the academic, psychological, physical, and social areas; and (v) studyholism antecedents, with a focus on the literature supporting the OCD-related conceptualization and the implications for the development of preventive and clinical interventions.

### 3.1. The Studyholism Inventories

To date, there are two instruments for evaluating studyholism—both of them designed within Loscalzo and Giannini’s [1] (internalizing) conceptualization: (i) the Studyholism Inventory (SI-10) [13,23], which allows for evaluating both studyholism and study engagement, and (ii) the Studyholism Inventory—Extended Version (SI-15) [24], which evaluates studyholism in its three components (obsessions, compulsions, and social impairment due to study). The SI-10 [13,23] is the first instrument developed by Loscalzo and colleagues [23] from a pool of 68 items that—based on the first definition by Loscalzo and Giannini [1]—covered addiction symptoms, obsessive-compulsive symptoms, and study engagement. As Loscalzo and Giannini [13] pointed out, the SI-10 has been created referring to the recommendations by scholars [29,33,42] regarding the analysis of new potential behavioral addictions and, more generally, new potential clinical conditions. After preliminary analyses on Italian college students, the SI-10 has been reduced to 10 items, with five items per two subscales: (i) Studyholism (comprehending obsessive-compulsive symptoms only) and (ii) Study Engagement (not comprehending vigor, dedication, and absorption items [30], but covering intrinsic motivation toward study) [23]. Next, Loscalzo and Giannini [13] deepened the analysis of the 10-item version of the test on a broader and more heterogeneous sample of Italian students. First, through Confirmatory Factor Analyses (CFAs), they found support for an 8-item version, which comprehends four items per subscale. Hence, they suggested including in the scoring of the SI-10 eight items (instead of 10). The fit indexes for this solution (reaching an improvement by allowing an error correlation) are good: GFI = 0.98 and RMSEA = 0.08. Concerning internal reliability, Cronbach’s alpha value is good for both the SI-10 subscales: 0.84 for Studyholism and 0.81 for Study Engagement [13]. Finally, Loscalzo and Giannini [13] supported the SI-10 convergent and divergent validity. More specifically, the SI-10 Studyholism subscale has a (medium) positive correlation with the BStAS and a (low) negative correlation with the Utrecht Work Engagement, short and student version (UWES-S-9; [43]). The SI-10 Study Engagement subscale has (medium) positive correlation with the UWES-S-9 and—in line with Atroszko et al.’s [9] findings—a low positive correlation with the BStAS [13]. Moreover, they proposed the cut-off scores for high and low studyholism/study engagement, which allows for screening Italian students for the four student types suggested by Loscalzo and Giannini [1]: engaged studyholics, disengaged studyholics, engaged students, and detached students. To calculate the cut-off, they looked for the raw scores corresponding to −1 and +1, that is, to the 40th and 60th T scores [13]. About these cut-off scores, Loscalzo and Giannini [13] pointed out that they could not perform a ROC analysis since studyholism is a new potential clinical condition; therefore, there were no available clinical sample and clinical instrument to be used as gold standards, which are needed to perform ROC analyses. However, these preliminary cut-off scores might be used to screen students and develop tailored interventions [13]. In this vein, Loscalzo and Giannini [13] proposed that different professional figures might use the SI-10 to evaluate if students encountered in their practice who face academic issues have high or low levels of studyholism and study engagement to tailor their intervention. Moreover, they pointed out how the SI-10 might be administered to all the students to detect those needing intervention [13].

Recently, the SI-10 has also been validated on Italian pre-adolescents and adolescents, confirming its good psychometric properties in younger students and providing the age-specific SI-10 cut-off scores for Italian students [44]. In line with the different developmental age groups, while the SI-10 fit indexes are similar in the pre-adolescent and adolescent samples, the cut-off scores differ among the two age groups [44]. Currently, the SI-10 has been translated—using the back-translation procedure—into the following languages, which are available to scholars upon request: English [45], Polish, Croatian, Spanish [46], Indonesian [47], and Chinese. 

The SI-15 [24] represents an extended assessment of studyholism through 11 more items than the SI-10 Studyholism subscale. It does not evaluate study engagement, but evaluates studyholism in its three components: (i) obsessions, already covered by the SI-10 Studyholism subscale (though the SI-15 has one more item than the SI-10); (ii) compulsions; and (iii) social impairment due to study. Academic impairment is not included since the opening questions included in the two Studyholism Inventories evaluate it [24]. The SI-15 arises from a pool of 45 items based on Loscalzo and Giannini’s [24] DSM-like tentative criteria, and it is made up of the three subscales mentioned above, each one composed of five items, for a total of 15 items in the final version of the test reached using Explorative and Confirmatory Factor Analyses. The SI-15 fit indexes—with the improvement reached by allowing three error correlations—are good: GFI = 0.95; RMSEA = 0.054. Cronbach’s alphas are good for the three subscales [Obsessions (0.85), Compulsions (0.82), and Social Impairment (0.86)] and for total score (0.89) that might be calculated given the moderate-to-high values of factor correlations [24]. Finally, Loscalzo and Giannini [24] supported the SI-15 convergent and divergent validity. More specifically, the SI-15 showed good divergent validity with the SI-10 Study Engagement subscale. In addition, the SI-15 showed moderate-to-high positive correlations with the SI-10 Studyholism subscale and good positive correlations with the BStAS [24].

Loscalzo and Giannini [24] highlighted that *the SI-15 might be considered an extended version of the SI-10 Studyholism subscale* since the SI-15 Obsessions subscale includes exactly the four items of the SI-10 Studyholism subscale, although these items were included among new ones. Hence, the SI-15 includes the SI-10 Studyholism subscale plus an additional 11 items addressing facets of studyholism (i.e., compulsions and functional impairment) not included in the SI-10. Therefore, they suggest using the SI-15 as a second step. The SI-10 is a quick screening instrument that distinguishes four types of students and detects those scoring high on studyholism. Those students should hence receive the SI-15 to extend their evaluation and be addressed in a clinical interview to consider their need for a clinical intervention [24]. The SI-15 is now available in languages other than Italian: English [45], Polish, and Spanish [46]. 

Finally, recently, Loscalzo et al. [45] analyzed the SI-10 and SI-15 measurement invariances between Italy and the USA, as well as between (rural) Western and (urban metropolitan) Southern U.S. students. Regarding the two U.S. groups, they found both metric and scalar measurement invariance for the SI-10, while they supported only metric invariance for the SI-15. The Italy–US comparison supported the metric invariance of the SI-15 but not of the SI-10. Therefore, they recommended further investigation of studyholism, considering context-specific factors across countries (and U.S. states) that might affect its interpretation and measurement.

*Highlight, n. 1:* In the literature, there are two instruments available for the assessment of studyholism: (i) the Studyholism Inventory (SI-10), which allows for evaluating both studyholism and study engagement and (ii) the Studyholism Inventory—Extended Version (SI-15), which evaluates obsessions, compulsions, and social impairment due to study. 

*Future Agenda, n. 1a:* Validating these instruments in other languages is critical to extending the analysis of studyholism across countries other than Italy, also considering the first results concerning their measurement invariance. 

*Future Agenda, n. 1b:* It is essential to adapt the SI-10 and the SI-15 for use with primary school children, including a parent-report version for children. 

*Future Agenda, n. 1c:* It would be valuable to employ ROC analyses to refine the SI-10 cut-off scores and define the SI-15 clinical cut-off scores.

### 3.2. Studyholism Prevalence 

The first study analyzing the spread of studyholism was by Loscalzo and Giannini [13]. After settling the SI-10 cut-off scores for high and low studyholism/study engagement, they screened their Italian college sample (n = 1296) and found that 10.8% of the students over-reached the cut-off score for high studyholism. Moreover, regarding the two types of studyholics, they found a higher spread of engaged studyholics (2.2%) than disengaged studyholics (1.7%). Later, Loscalzo [48] analyzed studyholism prevalence in a larger and more heterogeneous sample of Italian college students (n = 5159) and found an even higher spread of studyholism (15.4%). Concerning the two studyholic types, engaged studyholics (3.2%) are still more widespread than disengaged studyholics (2.2%). Another study conducted on Italian college students (n = 1223) reported the spread of high studyholism (11.8%) and confirmed the higher prevalence of engaged studyholics (1.7%) than disengaged studyholics (1.5%) [49]. Interestingly, another study on Italian college students (n = 422) reported a lower percentage of participants scoring high on studyholism (5.7%) compared to other studies [50]. Later, Loscalzo et al. [46] showed that high studyholism is also widespread among Spanish youths (13.1%), especially in the form of engaged studyholism (3.1%; disengaged studyholics = 1.0%). 

Then, regarding the U.S., Loscalzo et al. [45] analyzed the prevalence of studyholism and studyholic types in the merged sample of Western and Southern college students (given that the SI-10 factor means are invariant between the two samples). They found a lower percentage of youths with high studyholism (8.0%) than in Italy. However, similar to Italy, there is a higher spread of engaged studyholics (1.9%) than disengaged studyholics (0.8%). 

With regard to Eastern culture, in Saudi Arabia, Alshammari et al. [12] found that high studyholism was spread in their youth sample (15.31%), with a higher prevalence of engaged studyholics (5.92%) compared to disengaged studyholics (0.20%). Moreover, in Indonesian college students, Nugraha and Loscalzo [47] found a high spread of studyholism (11.5%) and engaged studyholism (5.7%). Interestingly, while Indonesian students showed a high percentage of engaged studyholism, there are no disengaged studyholics. Therefore, in the two Eastern countries analyzed so far, studyholism seems not to be an issue in its disengaged form. In contrast, there seems to be a higher prevalence of engaged studyholism compared to Western countries.

About younger students, Loscalzo et al. [44] found that in pre-adolescents, the spread of studyholism is critical, as they found that 18.6% of their sample (n = 451) over-reached the cut-off score for high studyholism (18.1% of the boys and 19.1% of the girls). In adolescents (n = 446), the prevalence is lower, even if still high: 11.9% (6.5% of boys and 16.3% of girls). In line with the differences in percentages between males and females in the age-specific samples, Loscalzo et al. [44] also highlighted a statistically significant interaction effect between gender and developmental age on studyholism and study engagement. For study engagement, their analyses showed that pre-adolescents have higher study engagement than adolescents. More specifically, boys and girls show decreased study engagement with the transition from pre-adolescence to adolescence (which is more evident in males than females). For studyholism, there is no difference in studyholism levels between pre-adolescents and adolescents. However, there is a gender-related difference concerning the developmental stage: boys have higher studyholism in pre-adolescence, while girls have higher studyholism in adolescence. Therefore, girls experience an increase in studyholism with the transition to adolescence, while boys experience a decrease from pre-adolescence to adolescence. Finally, regarding the spread of the two studyholic types, the prevalence of engaged studyholism is 2.0% for pre-adolescents and adolescents. It is higher than disengaged studyholism (1.6% and 1.8%, respectively, for the pre-adolescent and adolescent samples) [44]. Concerning adolescents, Loscalzo [51] reported a high percentage of 17.0% of students scoring high on studyholism in her sample (n = 793). In addition, engaged studyholics (3.4%) are more prevalent than disengaged studyholics (1.8%). Finally, in another study involving adolescents (n = 541), Loscalzo and Giannini [52] found a percentage of 13.3% for high studyholism and confirmed the higher prevalence of engaged studyholics (5.7%) compared to disengaged studyholics (0.9%). 

In conclusion, taking into account the limitations of the cut-off scores and the convenience sample used in the studies (as was also reported in a recent study about workaholism prevalence [53]), it might be suggested that studyholism has a higher prevalence in pre-adolescence compared to older ages (generally) since pre-adolescence represents the first critical transition for students, with likely associated distress, also due to higher cognitive demands. There is the passage from primary school to secondary school of first grade, and this school level corresponds to the one in which Italian students are required to decide on the study area to pursue through the next school level [54,55].

*Highlight, n. 2a:* Based on the paucity of research currently available, studyholism arose as a new potential clinical condition widespread in Italian students across different school levels (from secondary school of first level to university).

*Highlight, n. 2b:* Based on the few currently available studies, studyholism arose as widespread in countries other than Italy, including the U.S. and Eastern countries.

*Future Agenda, n. 2a:* The prevalence of studyholism needs to be addressed at the earliest stages of education, starting from primary school. This approach is crucial to identifying early indicators of studyholism, thereby enabling timely intervention and prevention interventions.

*Future Agenda, n. 2b*: Studyholism prevalence should be further addressed in other countries by considering the role that might be played by cultural factors (e.g., individualism versus collectivism).

### 3.3. Demographic and Study-Related Differences on Studyholism

Concerning *gender*, Loscalzo and Giannini [13] found that Italian women have higher studyholism than males. In the same line, Alshammari et al. [12] found that Saudi Arabian women score higher than men on studyholism. Then, Loscalzo [48] further specified that women have a (statistically significant) higher likelihood of being engaged studyholics (and detached students) than men. Instead, there is no gender-related difference in the spread of disengaged studyholics (and engaged students). Regarding younger students, Loscalzo et al. [44] highlighted no difference in studyholism levels between boys and girls in pre-adolescence. However, girls have higher studyholism than boys in adolescence. Finally, Loscalzo and Giannini [24], using the SI-15 on youths, showed that women have higher obsessions (and lower compulsions) than males. However, there is no difference in social impairment due to the study and the SI-15 total score. About younger students, it is interesting to note that Loscalzo et al. [44] stressed a statistically significant interaction effect between gender and developmental age on studyholism: girls experience an increase in studyholism with the transition to adolescence, while boys experience a decrease from pre-adolescence to adolescence. In summary, females (from adolescence) are more prone to studyholism in its obsessive/thinking component, while males (from adolescence) experience a decrease in obsessive-related studyholism but might be more prone to the compulsive/behavioral component at an older age.

Focusing on *age*, Loscalzo and Giannini [13] found no relationship between age and studyholism in college students. In the same vein, Loscalzo [48] did not find age-related differences among the four types of students and, therefore, between engaged and disengaged studyholics. Currently, no studies on adolescents addressed age-related differences or correlations between age and studyholism. Based on the available findings, it is possible to suggest that studyholism does not differ in age. However, there might be some differences concerning developmental age since Loscalzo et al. [44] highlighted the previously mentioned interaction between gender and developmental age in studyholism. 

Similarly, two studies analyzing the *school’s year* did not show differences among the different years across three school levels (i.e., the secondary school of first and second grade and university) [13,44], suggesting that studyholism should be targeted across all school years and from pre-adolescence, as there is no specific age or school year where it is higher. However, Loscalzo and Giannini [52] found that fifth-year adolescent students have higher studyholism than peers from other school years (except for second-year students). Alshammari et al. [12] found that fifth-year college students have higher studyholism than internship, first-year, and sixth-year students.

Regarding the *study area*, Loscalzo and Giannini [13] highlighted that students involved in humanities and educational majors (e.g., languages, literature, history, education) have higher studyholism than students from psychology and health professions courses. However, Loscalzo [48] did not find major-related differences between engaged and disengaged studyholics. Moreover, Loscalzo and Giannini [34]—using a path analysis model and a different coding of the various majors—did not find differences in studyholism. The only exception is for lower studyholism in humanities students, though the *β* value is near zero. Hence, based on the available evidence, there are no differences in studyholism among students enrolled in different college majors. Interestingly, there are instead some differences in adolescence. Loscalzo [51], using a path analysis model, found that the type of school does not predict studyholism. However, another study highlighted that adolescents attending a professional school have lower studyholism than peers attending a technical or a high school (with no difference found between technical and high school students) [44]. Similarly, Loscalzo and Giannini [52] found that professional school students have lower studyholism than students attending three different types of high school (i.e., classical, socioeconomic, and languages) and that there are no differences between the three high schools. Therefore, the available findings suggest that studyholism should be specially targeted in high schools, regardless of the specific study area.

Concerning other variables analyzed on college [13], pre-adolescent, and adolescent [44] students, to be involved (or not involved) in other activities besides studying (e.g., a sport or a hobby) does not affect studyholism levels across the three developmental ages. Regarding the variable about usually studying on weekends, adolescents and youths who answered yes to this question have higher studyholism than peers who do not study on weekends. For pre-adolescents, there is no difference. Finally, repeating a school year is associated with higher studyholism among college students only. 

In summary, these studies highlighted some demographic-related differences in studyholism. However, it should be noted that the SI-10 and SI-15 have yet to be tested for measurement invariance concerning these variables.

*Highlight, n. 3a:* Despite the limited research available, it is crucial to note that studyholism, particularly its obsessive component, is more prevalent in females from adolescence.

*Highlight, n. 3b:* Considering the few studies on the topic, males experience a decrease in studyholism levels from pre-adolescence to adolescence and are more prone to its compulsive component.

*Future Agenda, n. 3:* Studyholism should be evaluated through longitudinal studies (from childhood) to evaluate its developmental path, distinguishing between males and females and among the different studyholism components.

*Highlight, n. 4:* Based on the scant research available, studyholism levels do not differ based on age and—generally—school year.

*Future Agenda, n. 4:* Preventive interventions to reduce studyholism should be implemented at the earliest school level (possibly from childhood) across all school levels and school years.

*Highlight, n. 5:* As the first research showed, studyholism levels do not differ based on the area of study in youths, while in adolescence, studyholism is higher in High Schools.

*Future Agenda, n. 5:* Preventive interventions to reduce studyholism should be implemented, particularly in high schools, regardless of the specific study area.

*Highlight, n. 6:* The demographic-related differences must be read cautiously at present as there are no studies about measurement invariance regarding them.

*Future Agenda, n. 6:* A crucial step for future research is to analyze the SI-10 and SI-15 measurement invariances about demographic variables. This will provide further support to the differences observed in studyholism.

### 3.4. Studyholism Outcomes

The studyholism literature includes research about the negative downsides associated with studyholism in both adolescents and youths across different functional areas: academic, psychological, physical, and social. 

#### 3.4.1. Academic Outcomes

Regarding academic-related outcomes, there is research on Grade Point Average (GPA), time spent studying, number of exams given, dropout intention, and academic resilience. Concerning *GPA* and *time spent studying*, Loscalzo and Giannini [13] found a negative and low (−0.19) correlation between studyholism and GPA. Moreover, there is a positive (even if still low, as *r* values range between 0.10 and 0.17) correlation of studyholism with time spent studying (self-reported hours of study per day and days of study per week, distinguishing between general attitude and the preparation for a test). Similarly, a path analysis model on Italian college students showed that studyholism is a (weak) positive predictor of hours spent studying daily (generally and before exams). Studyholism has instead been found to negatively predict GPA, though the beta value is low (−0.08) [34]. Loscalzo and Giannini [24], using the SI-15, highlighted that GPA is negatively (and weakly) correlated with the Obsessions subscale. In contrast, it positively correlates with Compulsions and Social Impairment subscales (no correlation with the total score). Regarding time spent studying (evaluated through the four variables used by Loscalzo and Giannini [13]), they found that it is positively correlated with all three SI-15 subscales and the total score (values ranging between 0.18 and 0.37) [24]. Similarly, in Spanish college students [46], the correlations between time spent studying and both the SI-10 Studyholism subscale and the SI-15 subscales are all positive (ranging between 0.15 and 0.28 except for a lack of correlation between Social Impairment and days per week of studying before exams). Regarding GPA, it has a low negative correlation with the SI-10 Studyholism and the SI-15 Obsessions subscales and a low positive correlation with the SI-15 Compulsions subscale. Finally, the path analysis conducted on Italian adolescents showed that studyholism does not predict GPA, while it positively predicts time spent studying (hours per day of studying generally and before a school test) [51]. Finally, a study conducted on Indonesian college students showed that studyholism is a low (negative) predictor of GPA, while it does not predict the hours of study per day generally and before exams [47]. 

Related to academic performance, Loscalzo and Giannini [49] also analyzed the *number of exams given* (regardless of the outcome) in the last examination session. They found that the SI-10 Studyholism subscale positively predicts this variable (*β* = 0.20); however, when using the SI-15, only the Compulsions subscale is found to predict the number of exams given (*β* = 0.15), which is in line with Loscalzo and Giannini’s findings [24] that it is the compulsive component of studyholism to be associated with a higher GPA, and not the obsessive one (which is not a predictor or is a low negative predictor of GPA). 

Finally, with regard to academic-related outcomes, two studies concerning *dropout intention* showed that studyholism positively predicts the intention to give up studies in both adolescents [51] and college [34] Italian students, with a *β* value, respectively, of 0.32 and 0.27. There is only one study about *academic resilience* [47]. Studyholism predicts a worse academic resilience: it does not predict reflective adaptive help-seeking and perseverance, while it is a positive predictor of negative affect and emotional response [47]. Summarizing the key findings, the research on academic outcomes reveals that studyholism is consistently linked to increased study time across all educational levels analyzed. However, the relationship between studyholism and GPA is not straightforward, suggesting that higher study time does not always translate to better grades. Additionally, two studies indicate that studyholism is associated with a higher intention to dropout, while another study highlights a negative impact on academic resilience, potentially leading to early school dropout.

*Highlight, n. 7:* The paucity of current research suggests that studyholism is associated with academic impairment and dropout intention in both adolescents and college students.

*Future Agenda, n. 7:* Preventive interventions to detect the risk of studyholism should be implemented as soon as possible since it might be associated with early school dropout, especially in adolescents.

#### 3.4.2. Psychological, Physical, and Social Outcomes

Concerning the studyholism literature about *psychological outcomes*, Loscalzo and Giannini [34] and Loscalzo [51] showed that studyholism is a positive predictor of negative affect and general stress, with a higher impact on college students compared to adolescents, thus suggesting that studyholism leads to a higher psychological impairment in youths compared to adolescents. Moreover, Molinaro et al. [56] and Sanseverino et al. [57] highlighted that studyholism is associated with higher academic exhaustion. Regarding psychopathology, Loscalzo and Giannini [49] found that studyholism is associated with worse mental health, as the SI-10 Studyholism subscale and the SI-15 Obsessions subscale are positive predictors of all the nine internalizing and externalizing symptoms they analyzed (i.e., somatization, obsessive-compulsive, interpersonal sensitivity, depression, anxiety, hostility, phobic anxiety, paranoid ideation, and psychoticism). Moreover, a study about defense mechanisms highlighted that students characterized by high studyholism score higher on the maladaptive style compared to students having low studyholism; more specifically, they score higher on these defense mechanisms: acting-out, projective identification, help-rejecting complaining, projection, regression, withdrawal, splitting, and somatization [50]. Finally, a study on adolescents highlighted that those characterized by high studyholism—compared to peers with low studyholism levels—have higher social anxiety [52].

Regarding *physical outcomes*, the studyholism literature shows that it is associated with worse physical health since studyholism arose as a positive predictor of sleep quality impairment and daytime sleepiness in both youths [34] and adolescents [51], as well as of insomnia in college students [12,56]. Finally, regarding *social outcomes*, studyholism positively predicts family and friends’ complaints due to overstudying in college students [34], as well as social relationship impairment due to studying in both youths [34] and adolescents [51]. Moreover, adolescents with high studyholism have a higher tendency to interpret (ambiguous) social situations negatively compared to their peers with low studyholism [52].

*Highlight, n. 8:* Even if considering the few existing studies on the topic, studyholism is associated with psychological, physical, and social impairment in both adolescents and college students.

*Future Agenda, n. 8:* Preventive and clinical interventions addressing studyholism are imperative to avoid functional impairment in students.

### 3.5. Studyholism Antecedents

In the studyholism literature, there are papers about studyholism antecedents, which have been analyzed to shed light on potential targets for preventive and clinical interventions and to test whether studyholism might be defined as an OCD-related disorder (or, more generally, as an internalizing rather than externalizing disorder), as Loscalzo and Giannini suggested, e.g., [13,24,31]. 

#### 3.5.1. Studies Supporting the OCD-Related (or Internalizing) Conceptualization of Studyholism

First, Loscalzo and Giannini [34] and Loscalzo [51] analyzed a few potential antecedents simultaneously: study-related perfectionism, perfectionistic strivings, perfectionistic concerns, and trait worry (for individual antecedents), and parents’ overstudy climate, teachers’ overstudy climate, and study area (for situational antecedents). The path analysis model showed that, among college students, only one statistically significant predictor reaches the selected cut-off of 0.10 for the *β* value: trait worry (*β* = 0.67) [34]. In adolescents, trait worry is still the strongest predictor (*β* = 0.62); moreover, teachers’ overstudy climate reaches the cut-off of 0.10; however, the *β* value is still meager [51]. These two studies, highlighting that *trait worry* is a strong predictor of studyholism, provided support to the OCD-related conceptualization since the previous literature showed that trait worry is a transdiagnostic process across internalizing disorders [58,59], also contributing to OCD [60]. 

Then, Loscalzo and Giannini [49] analyzed *psychopathology* (internalizing and externalizing symptoms) and *sensation seeking* (evaluated in the following dimensions: thrill and adventure seeking, experience seeking, disinhibition, and boredom susceptibility) in college students. They found that the SI-10 Studyholism subscale is positively predicted by obsessive-compulsive, depression, and anxiety (i.e., internalizing symptoms) and negatively predicted by psychoticism and boredom susceptibility (i.e., externalizing features), hence supporting the OCD-related conceptualization (against the behavioral addiction theorization). In the same line, the path analysis model conducted using the SI-15—hence differentiating between the three studyholism components—highlighted similar predictors, and, most notably, the SI-15 Obsessions subscale is the only SI-15 subscale to be explained by the predictors in a good percentage of variance. Moreover, the SI-15 Compulsions subscale is predicted positively by anxiety and negatively by hostility and disinhibition. Therefore, considering that compulsions characterize both OCD and addictions, Loscalzo and Giannini [49] speculated that this result further supports that problematic overstudying is better conceptualized as an OCD-related disorder rather than an addiction since even the compulsion component is positively predicted by internalizing features and negatively by externalizing ones. This study provides additional support to the OCD-related conceptualization through the results that arose from the path analysis including psychopathology as an outcome. In fact, Loscalzo and Giannini [49] pointed out that the psychopathology scale whose variance is explained the most by their model (with studyholism and study engagement as predictors of psychopathology) is the Obsessive-Compulsive scale (followed by depression and anxiety scales, which are internalizing scales). Finally, the path analysis model performed using the SI-15 highlighted that it is the obsessive component (compared to the compulsive one) of studyholism that is critical in predicting worse mental health and academic performance [49].

Some support for the OCD-related conceptualization also came from Loscalzo and Giannini’s [50] study concerning *defense mechanisms*. In summary, from the maladaptive cluster, regression, projective identification, help-rejecting complaining, withdrawal, and somatization are positive predictors. Also, from the image-distorting style, omnipotence is a negative predictor. Finally, suppression arises as a negative predictor among the adaptive defenses, while task-orientation and pseudo-altruism are positive predictors. Other defense mechanisms predicting studyholism (though with low *β* values) are anticipation (positive predictor), affiliation, and sublimation (negative predictors). 

Besides supporting the definition of problematic overstudying as a clinical condition—given that the strongest positive predictor of studyholism is the maladaptive defense of *regression*—a critical analysis made by the authors highlighted that most of the defense mechanisms predicting studyholism are in line with the OCD-related framework. Concerning the defense mechanisms explained by Loscalzo and Giannini [50] with a specific focus on their role in supporting the OCD-related conceptualization, *suppression*—reflecting the ability to control thoughts—arose as a negative predictor of studyholism, in line with its obsessive nature. About *task-orientation* (i.e., working heavily to cope with stress), this is instead a positive predictor in line with the conceptualization of the over-studying behavior as the compulsion put in place by the students to cope with their distressing feelings/thoughts. For *anticipation* (e.g., thinking about and planning an exam, trying to predict a negative situation to cope with it), it is a positive predictor in line with the obsessive thinking posed by the OCD-related framework. Finally, studyholism is negatively predicted by *omnipotence* and *sublimation*, in contrast with the literature about the defense mechanisms characterizing substance use disorders [61] and in line with studies highlighting that lower sublimation and higher regression—which is the strongest predictor of studyholism—is associated with OCD [62,63].

Another study, which has been conducted on adolescents, focused on social anxiety and interpretation bias as potential antecedents of studyholism [52]. Among the main findings, *social anxiety* arose as a strong positive predictor of studyholism (*β* = 0.40), in line with the internalizing (rather than externalizing) conceptualization; moreover, a *negative interpretation style in non-social ambiguous situations* characterizes studyholism, as it is positively predicted by the tendency to interpret these situations negatively or neutrally (rather than positively). Given that studyholism is not predicted by a negative interpretation bias in social situations—which is typical of social anxiety [64,65]—this supports that social anxiety and studyholism are two different diagnoses (even if studyholism is positively predicted by social anxiety) in line with the DMS-5-like tentative criteria of studyholism, which underscore that a diagnosis of studyholism should not be posed if another clinical diagnosis explains the overstudying behavior [24]. Loscalzo and Giannini [52] also found that adolescents with high studyholism, when facing ambiguous non-social situations, tend to have a greater likelihood of having all possible interpretations (positive, negative, and neutral) popping up in their minds. However, they do not have a higher probability of selecting the negative interpretation as the most believable. Therefore, Loscalzo and Giannini [52] suggest that this might align with the obsessive nature of studyholism, as students might overthink/ruminate about non-social situations without concluding how to interpret that event. Similarly, Molinaro et al. [56] also supported the OCD-related conceptualization of studyholism, given their findings that studyholism positively predicts insomnia; they suggest that studyholics might ruminate about academic-related topics, developing, in turn, sleep problems.

Another study conducted by Loscalzo and Giannini [66] gave further support to the OCD-related conceptualization by focusing on *intolerance for uncertainty* as a predictor of studyholism in youths. In fact, they found that it is a positive predictor (*β* = 0.36), in line with the presence of this feature across different internalizing disorders, including OCD [67,68,69,70]. Finally, even if not related to studyholism antecedents, the study by Loscalzo et al. [44] should be mentioned as providing support to the OCD-related conceptualization through the analysis of the interaction between the developmental level (i.e., pre-adolescence and adolescence) and gender on studyholism. More specifically, they found that females experience an increase in studyholism levels with age, while males experience a decrease. Therefore, while boys have higher studyholism in pre-adolescence, girls report higher studyholism in adolescence. In fact, Loscalzo et al. [44] also found a similar prevalence of studyholism across genders in pre-adolescence but a higher prevalence in females in adolescence. This developmental trend is in line with the feature of OCD, which is usually more prevalent in females in adulthood, even if males are characterized by an earlier onset [35]. Moreover, it is in contrast with the addiction literature, which shows that substance use disorders and gambling disorder are more prevalent in males than in females [35].

*Highlight, n. 9:* There is research on studyholism supporting the OCD-related framework. 

*Future Agenda, n. 9:* Further studies should be conducted to shed more light on the OCD-related nature of studyholism, especially analyzing its cerebral correlates.

#### 3.5.2. Implications for Preventive and Clinical Interventions

The literature about studyholism antecedents provided valuable insights concerning how to develop preventive and clinical interventions. 

First, given the substantial positive predictive value of trait worry on studyholism in youths [34] and adolescents [51], this variable stands out as the primary focus of interventions to prevent or reduce studyholism. Therefore, Loscalzo and Giannini [34] suggest that programs already tested for reducing trait worry might be applied in the school context. 

Then, referring to the role of social anxiety in predicting studyholism, Loscalzo and Giannini [52] indicated the value of screening for studyholism adolescents displaying high social anxiety. Moreover, they suggested that previously validated interventions for social anxiety might be tested on studyholics as clinical and preventive interventions. Additionally, concerning the results related to the interpretation style characterizing studyholics in social and non-social situations, they proposed that clinical interventions aimed at reducing studyholism should target non-social situations by decreasing the tendency to interpret these situations negatively or neutrally (and favoring the positive interpretation), with a specific focus on school performance and situations. Finally, based on their result that high school is the school type where studyholism is more widespread (compared to professional and technical schools), Loscalzo and Giannini [52] suggest that, even if preventive interventions are critical for all school levels and school types, they are especially needed in high schools.

Moreover, Loscalzo and Giannini [50] pointed out that, given the critical role of defense mechanisms in predicting studyholism, psychodynamic therapies (e.g., Freudian therapy, Jungian therapy, and Sandplay therapy) might be applied to reduce studyholism.

Finally, some studies supported the value of distinguishing between engaged and disengaged studyholics, as they have different relationships with some antecedents and outcomes [34,49,51,52]. Therefore, it is critical to tailor preventive and clinical interventions on the studyholic type. Also, Loscalzo [51] highlighted the critical value of preventing studyholism through interventions implemented even during primary school since her study showed that studyholism during adolescence leads to weaker psychological and social impairment compared to youths. Therefore, detecting early risk indicators of studyholism from childhood would be necessary.

*Highlight, n. 10:* The current literature suggests that interventions targeting worry, social anxiety, and the interpretation bias in non-social situations, as well as psychodynamic therapies, might be effective in preventing and treating studyholism. 

*Future Agenda, n. 10:* Future studies should test such interventions across different ages to test their efficacy for studyholism.

## 4. Conclusions

In 2017, Loscalzo and Giannini [1] introduced a comprehensive conceptualization of the new construct of studyholism (or obsession toward studying) in the scientific literature. This conceptualization was based on their theoretical reflections and preliminary research data, and they advocated for subsequent research that could have provided evidence for the appropriateness of the OCD-related (or, more generally, internalizing) conceptualization. Seven years after this publication, growing research supported Loscalzo and Giannini’s theorization of studyholism as an OCD-related disorder. However, scholars have also used the term “studyholism” to refer to different measures and/or theoretical definitions [10,11,12]. Therefore, this paper reviewed the studyholism literature to systematize the findings and avoid confusion surrounding this new potential clinical condition. Thus, after presenting Loscalzo and Giannini’s [1] conceptualization and highlighting the difference between studyholism and study addiction [9], this paper introduced the findings from the studies published so far. More specifically, besides presenting the studyholism inventories and the studyholism prevalence, this paper reported the demographic and study-related differences in studyholism, studyholism outcomes (distinguishing among the academic, psychological, physical, and social areas), and the studyholism antecedents. In the last section about antecedents, the literature concerning the variables that arose as significant predictors of studyholism has been presented in light of the implications for supporting OCD-related conceptualization and developing preventive and clinical interventions. Finally, for each section, a research agenda has been suggested.

Regarding the limitations of the current review, it is necessary to highlight that the authors of the construct have mainly conducted research on studyholism (except for a few cases), and this might favor a confirmation bias. Therefore, increasing the research on studyholism among other research groups is critical. Additionally, the theorization of studyholism has been better defined based on the preliminary psychometric data on this scale (that included addiction items in the initial pool of items [23]). However, subsequent research has later been conducted using the SI-10 and SI-15, which are instruments developed in line with the OCD-related conceptualization, and which might limit the generalizability of the findings due to the lack of robust external validation. Finally, the accuracy of the prevalence and impact of studyholism might be affected by the scales being self-report measures. 

Among the main points highlighted by this review, future studies should extend the analysis of studyholism across different countries and from primary school to detect early indicators of studyholism and implement timely preventive interventions, which are recommended across all school levels and school years, especially in light of the functional impairment associated with studyholism across different areas which includes a higher risk for early school dropout. Moreover, there is evidence concerning trait worry, social anxiety, and interpretation bias for non-social situations as potential targets for preventing and treating studyholism and the potential for psychodynamic therapies to help reduce studyholism. Therefore, it is critical to test these interventions for efficacy. Finally, despite the research evidence gathered until now supporting the OCD-related conceptualization of studyholism, studies about the cerebral correlates of problematic overstudying would be critical in further unveiling the internalizing and/or externalizing nature of this new potential clinical condition. As previously pointed out by Loscalzo and Giannini [31], OCD is associated with dysfunctions in the orbitofrontal cortex, anterior cingulated cortex, and striatum, while Gambling Disorder (i.e., a behavioral addiction) is characterized by the activation of the brain reward system (like for substance disorders), and substance disorders are also associated with changes in the brain circuits. Therefore, finding cerebral features similar to OCD or addictions in problematic overstudying might give some insights into the proper definition of this new potential clinical condition as an internalizing and/or externalizing disorder.

Finally, keeping in mind that this review is based on the studyholism literature but that there is another theoretical perspective about problematic overstudying (i.e., study addiction), future research must deepen the analysis of problematic overstudying using different perspectives to offer new insights into the proper conceptualization of this new potential clinical disorder associated with overstudying.

## Data Availability

Not applicable.
